# Protein N-myristoylation plays a critical role in the mitochondrial localization of human mitochondrial complex I accessory subunit NDUFB7

**DOI:** 10.1038/s41598-023-50390-z

**Published:** 2023-12-27

**Authors:** Haruna Harada, Koko Moriya, Hirotsugu Kobuchi, Naotada Ishihara, Toshihiko Utsumi

**Affiliations:** 1 Graduate School of Sciences and Technology for Innovation, Yamaguchi University, Yamaguchi, Japan; 2 Department of Biological Chemistry, Faculty of Agriculture, Yamaguchi University, Yamaguchi, Japan; 3 Department of Biological Sciences, Graduate School of Science, Osaka University, Osaka, Japan; 4 Department of Cell Chemistry, Dentistry and Pharmaceutical Sciences, Okayama University Graduate School of Medicine, Okayama, Japan

**Keywords:** Biochemistry, Cell biology, Chemical biology, Molecular biology

## Abstract

The present study examined human N-myristoylated proteins that specifically localize to mitochondria among the 1,705 human genes listed in MitoProteome, a mitochondrial protein database. We herein employed a strategy utilizing cellular metabolic labeling with a bioorthogonal myristic acid analog in transfected COS-1 cells established in our previous studies. Four proteins, DMAC1, HCCS, NDUFB7, and PLGRKT, were identified as N-myristoylated proteins that specifically localize to mitochondria. Among these proteins, DMAC1 and NDUFB7 play critical roles in the assembly of complex I of the mitochondrial respiratory chain. DMAC1 functions as an assembly factor, and NDUFB7 is an accessory subunit of complex I. An analysis of the intracellular localization of non-myristoylatable G2A mutants revealed that protein N-myristoylation occurring on NDUFB7 was important for the mitochondrial localization of this protein. Furthermore, an analysis of the role of the CHCH domain in NDUFB7 using Cys to Ser mutants revealed that it was essential for the mitochondrial localization of NDUFB7. Therefore, the present results showed that NDUFB7, a vital component of human mitochondrial complex I, was N-myristoylated, and protein N-myrisotylation and the CHCH domain were both indispensable for the specific targeting and localization of NDUFB7 to mitochondria.

## Introduction

Protein N-myristoylation is an important fatty acylation of proteins in eukaryotes and viruses. In this modification, myristic acid is covalently bound to the α-amino group of the N-terminal Gly of proteins through an amido bond after the removal of the initiating Met by methionine aminopeptidase^1–4^. Therefore, protein N-myristoylation occurs cotranslationally on newly synthesized proteins. In addition to cotranslational N-myristoylation, posttranslational N-myristoylation was found to occur on many caspase-cleavage products on an internal Gly exposed by caspase cleavage during apoptosis^5–7^. N-myristoyltransferase, a member of the GCN5-related *N*-acetyltransferase superfamily of proteins^8^, catalyzes both cotranslational and posttranslational N-myristoylation. Many N-myristoylated proteins play essential roles in the regulation of a wide variety of signal transduction pathways in cells, and include guanine nucleotide-binding proteins, Ca^2+^-binding proteins, protein kinases and their substrates, phosphatases, E3-ubiquitin ligases, and apoptosis-related proteins. Besides these cellular signal transduction proteins, protein N-myristoylation is found on many disease-associated proteins^9–13^. In many cases, reversible membrane binding mediated by protein N-myristoylation regulates the functions of these proteins. Therefore, protein N-myristoylation is regarded as a lipid modification that mainly occurs on cytoplasmic soluble proteins; however, some N-myristoylated proteins that specifically localized to intracellular organelles were also found. We previously established a strategy to comprehensively identify human N*-*myristoylated proteins from human cDNA clones in human cDNA resources. In this strategy, N-myristoylated proteins were detected by metabolic labeling and mass spectrometric analyses of proteins obtained by an in vitro translation system^14–16^.

Using this strategy, we identified two mitochondrial membrane proteins, TOMM40 and SAMM50, as N-myristoylated membrane proteins^17,18^. Both of these proteins are β-barrel proteins that localize to the outer membrane of mitochondria. TOMM40 is a major component of the translocase of the mitochondrial outer membrane (TOM complex) that acts as a general import pore for most mitochondrial precursor proteins^19,20^. SAMM50 is a major component of the SAM complex required for the sorting and assembly of β-barrel proteins^21^. An analysis of the intracellular localization of non-myristoylatable G2A mutants revealed that protein N-myristoylation occurring on SAMM50, but not on TOMM40, plays a critical role in the mitochondrial targeting of this protein^18^. However, the function of protein N-myristoylation occurring on TOMM40 remains unclear. Based on these findings, protein N-myristoylation may mediate diverse roles in the intracellular localization and function of mitochondrial proteins. In the present study, we searched for human N-myristoylated proteins that specifically localize to mitochondria among the 1,705 human genes listed in MitoProteome, a mitochondrial protein database^22^. The results obtained revealed that four proteins, DMAC1, HCCS, NDUFB7, and PLGRKT, were N-myristoylated proteins that specifically localized to mitochondria. Among these proteins, DMAC1 and NDUFB7 were previously found to play critical roles in the assembly of complex I of the mitochondrial respiratory chain as an assembly factor and an accessory subunit, respectively^23^. An analysis of the intracellular localization of non-myristoylatable G2A mutants revealed that protein N-myristoylation occurring on NDUFB7 played a critical role in the mitochondrial localization of this protein. Furthermore, an analysis of the role of the CHCH domain (CHCHD) found in NDUFB7 using Cys to Ser mutants revealed that CHCHD was vital for the mitochondrial localization of NDUFB7. Therefore, the present results showed that NDUFB7, an essential component of human mitochondrial complex I, was N-myristoylated, and protein N-myristoylation and CHCHD were both indispensable for the specific targeting and localization of NDUFB7 to mitochondria.

## Results

### Identification of human N-myristoylated mitochondrial proteins from human genes listed in the MitoProteome protein database

**Figure 1:**
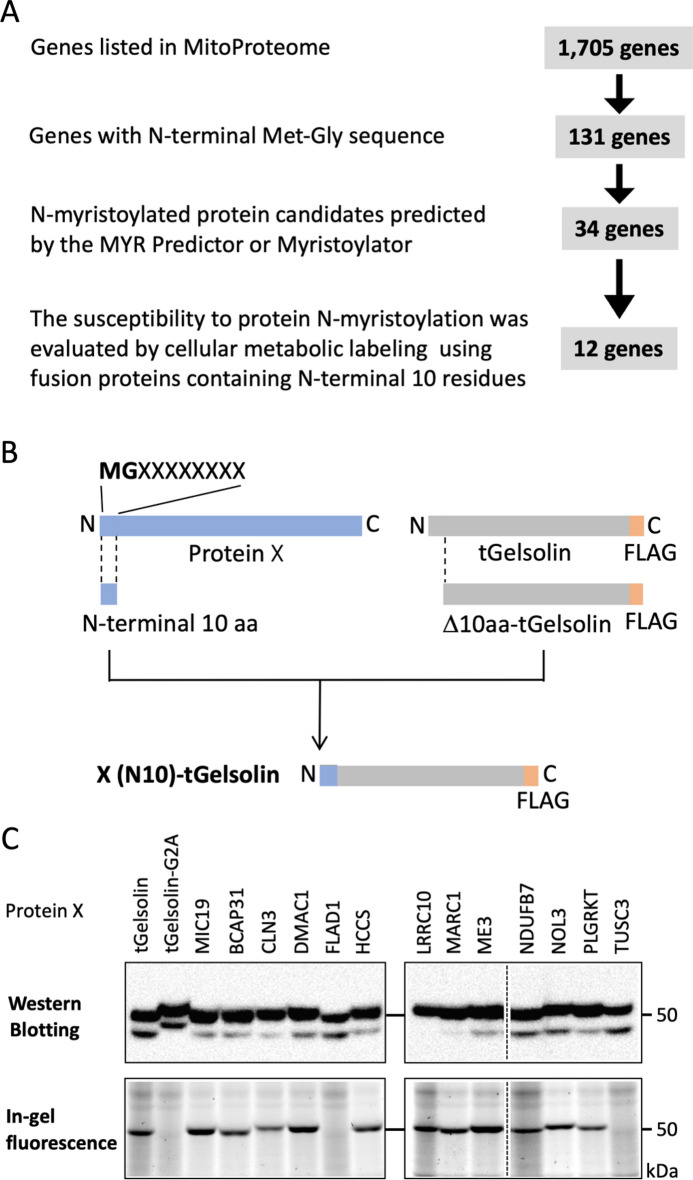
Identification of N-myristoylated mitochondrial proteins from human genes listed in the MitoProteome protein database. Twelve candidate genes (*BCAP31, CLN3, DMAC1/TMEM261, FLAD1, HCCS, LRRC10, MARC1/MTARC1, ME3, NDUFB7, NOL3, PLGRKT and TUSC3*) were selected as N-myristoylated mitochondrial proteins from the human genes listed in MitoProteome, a mitochondrial protein database, and their susceptibility to protein N-myristoylation was evaluated by in vivo metabolic labeling in transfected COS-1 cells using fusion proteins in which the 10 N-terminal amino acid residues were fused to the FLAG-tagged model protein, tGelsolin. (**A**) Strategy to identify N-myristoylated mitochondrial proteins from human genes listed in the MitoProteome protein database. (**B**) Schematic representation of the generation of X(N10)-tGelsolin-FLAG with 10 N-terminal amino acids of the candidate protein X at its N terminus. (**C**) Analysis of the protein N-myristoylation of X(N10)-tGelsolin-FLAGs by metabolic labeling in transfected COS-1 cells. cDNAs coding X(N10)-tGelsolin-FLAGs were transfected into COS-1 cells. The expression of proteins was evaluated by Western blotting using an anti-FLAG antibody (upper panels). Protein N-myristoylation was evaluated by metabolic labeling with a myristic acid analog followed by click chemistry, as described in the Methods (lower panels). Images obtained by Western blotting or metabolic labeling were cropped. Uncropped full-length raw image data are shown in Supplementary Figure S1.

We searched for human N-myristoylated mitochondrial proteins among the human genes listed in MitoProteome, a mitochondrial protein database, using a strategy established in our previous studies^14–18^. In this strategy, 131 genes with N-terminal Met-Gly motifs were extracted from the 1,705 human genes listed in the MitoProteome protein database^22^. After applying the N-terminal sequences of the proteins encoded by these genes to two protein N-myristoylation prediction programs, the MYR Predictor and Myristoylator^24,25^, 34 positively predicted genes were identified (Supplementary Table S1). Among these positively predicted genes, 12 candidate genes (*BCAP31, CLN3, DMAC1/TMEM261, FLAD1, HCCS, LRRC10, MARC1/MTARC1, ME3, NDUFB7, NOL3, PLGRKT and TUSC3*) for N-myristoylated mitochondrial proteins were selected and their susceptibility to protein N-myristoylation was evaluated using fusion proteins in which the 10 N-terminal amino acid residues were fused to a FLAG-tagged model protein, tGelsolin, as previously described^14^ (Fig. 1A,B). The information about the 13 genes analyzed in this study was summarized in Supplementary Table S2.

In this experiment, N-myristoylatable tGelsolin-FLAG, its non-N-myristoylatable mutant (tGelsolin-G2A-FLAG), and the tGelsolin fusion protein of MIC19 (CHCHD3), a known N-myristoylated mitochondrial protein, were used as control proteins. To establish whether these fusion proteins are N-myristoylated, cellular metabolic labeling experiments on COS-1 cells using Alk-Myr, a bioorthogonal myristic acid analog, were performed^16–18^. In these experiments, after labeling cells transfected with cDNA coding C-terminally FLAG-tagged tGelsolin-fusion proteins with Alk-Myr, the N-myristoylated protein was detected with click chemistry. As shown in the upper panels of Fig. 1C, efficient protein expression in COS-1 cells was equally observed for all the fusion proteins tested, including 3 control proteins, according to a Western blotting analysis. Metabolic labeling experiments revealed the efficient incorporation of Alk-Myr into the fusion proteins of 10 candidate genes (*BCAP31, CLN3, DMAC1/TMEM261, HCCS, LRRC10, MARC1/MTARC1, ME3, NDUFB7, NOL3, and PLGRKT*), similar to two N-myristoylatable control proteins (tGelsolin-FLAG and MIC19(N10)-tGelsolin-FLAG); however, this incorporation was not observed in the 2 fusion proteins of 2 candidate genes (*FLAD1* and *TUSC3*), as was the case for the non-N-myristoylatable tGelsolin-G2A mutant (Fig. 1C, lower panels).

**Figure 2:**
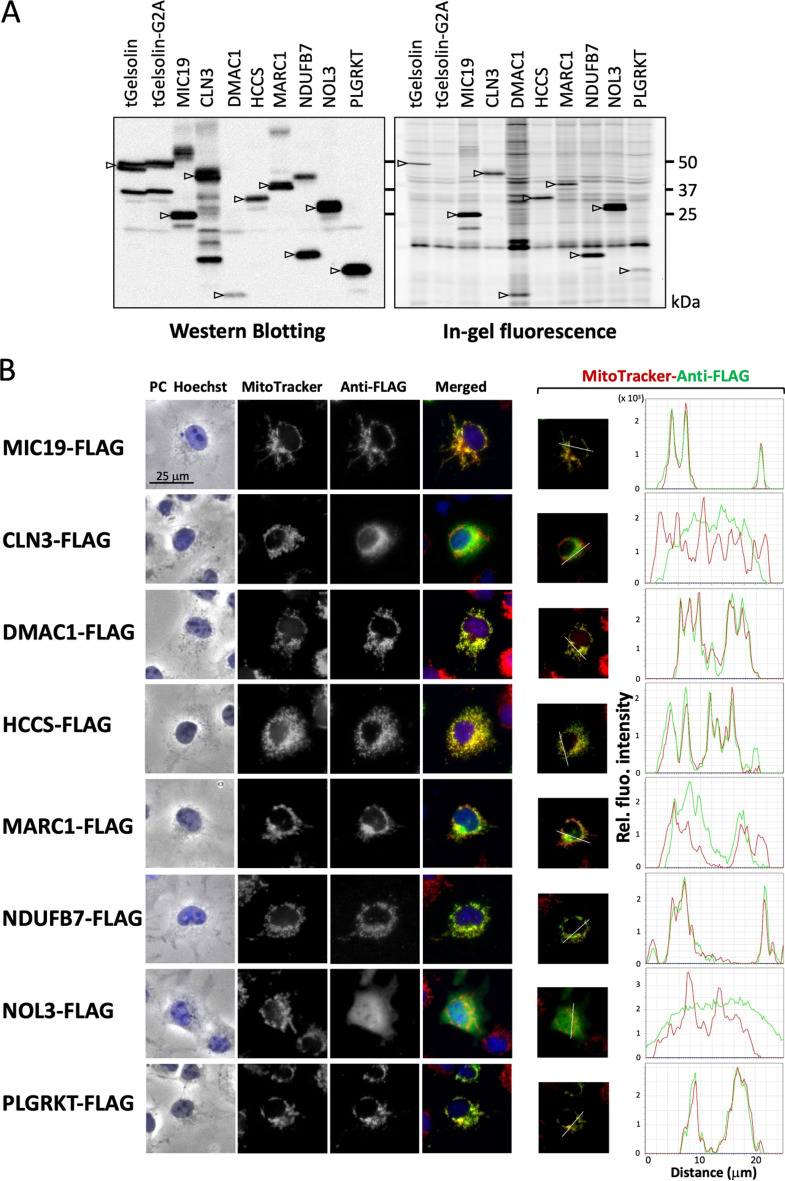
DMAC1, HCCS, NDUFB7, and PLGRKT are N-myristoylated mitochondrial proteins. Seven proteins (CLN3, DMAC1, HCCS, MARC1, NDUFB7, NOL3, and PLGRKT) were selected from proteins in which protein N-myristoylation was detected in X(N10)-tGelsolin-FLAG, and their protein N-myristoylation and intracellular localization were evaluated using C-terminally FLAG-tagged full-length proteins. (**A**) Analysis of the protein N-myristoylation of CLN3, DMAC1, HCCS, MARC1, NDUFB7, NOL3, and PLGRKT expressed in transfected COS-1 cells by metabolic labeling. cDNAs coding C-terminally FLAG-tagged full-length proteins were transfected into COS-1 cells and cells were then labeled with a myristic acid analog. The expression of proteins was evaluated by Western blotting using an anti-FLAG antibody (left panel). Protein N-myristoylation was evaluated by metabolic labeling followed by click chemistry, as described in the Methods (right panel). Arrows indicate the position of the expressed proteins. The images obtained by Western blotting or metabolic labeling were cropped. Uncropped full-length raw image data are shown in Supplementary Figure S2. (**B**) Analysis of the intracellular localization of CLN3, DMAC1, HCCS, MARC1, NDUFB7, NOL3, and PLGRKT by an immunofluorescence microscopic analysis. The intracellular localization of seven proteins was assessed by an immunofluorescence analysis of COS-1 cells transfected with cDNA coding C-terminally FLAG-tagged full-length proteins using an anti-FLAG antibody. MIC19-FLAG was used as a control mitochondrial N-myristoylated protein. Hoechst and MitoTracker Red were used as organelle markers for the nucleus and mitochondria, respectively. Experiments were repeated 3 times and similar results were obtained. Representative data are shown. Abbreviation used: PC, phase contrast image. The results of the line profile analysis are shown. In the line profile analysis, corresponding line-scan graphs of the relative fluorescence intensities of green anti-FLAG fluorescence (green line) and red MitoTracker red fluorescence (red line) along the white line indicated in the merged images are shown.

To clarify whether the full-length protein was N-myristoylated, we selected 7 genes (*CLN3*, *DMAC1/TMEM261, HCCS, MARC1/MTARC1*, NDUFB7, *NOL3, and PLGRKT*) and their susceptibility to protein N-myristoylation was evaluated using the same strategy with the full-length cDNA coding for the C-terminally FLAG-tagged protein. The results obtained are shown in Fig. 2A.

In the left panel, all proteins, including three control proteins (tGelsolin-FLAG, tGelsolin-G2A-FLAG, and MIC19-FLAG), were expressed based on a Western blotting analysis using an anti-FLAG antibody. Metabolic labeling experiments revealed the incorporation of Alk-Myr in all 7 proteins encoded by the 7 tested genes, similar to the two N-myristoylatable control proteins (tGelsolin-FLAG and MIC19-FLAG), as shown in Fig. 2A right panel, indicating that the 7 proteins encoded by the 7 tested genes (*CLN3*, *DMAC1/TMEM261, HCCS, MARC1/MTARC1*, *NDUFB7, NOL3, and PLGRKT*) were N-myristoylated proteins. The characteristics of these proteins are summarized in Table 1.

**Table 1:** Proteins found to be N-myristoylated in the present study.

Accession no	Protein name	Gene name	Length (aa)	Protein function	Reference
Q13286	Ceroid-lipofuscinosis, neuronal 3	*CLN3*	438	Mediates microtubule-dependent, anterograde transport connecting the Golgi network, endosomes, autophagosomes, lysosomes, and plasma membrane	^26^
Q96GE9	Distal membrane-arm assembly complex protein 1	*DMAC1/TMEM261*	116	Assembly factor of the mitochondrial NADH:ubiquinone oxidoreductase complex (Complex I)	^23^
P53701	Holocytochrome c synthase	*HCCS*	268	Lyase that catalyzes the covalent linking of the heme group to the cytochrome C apoprotein in order to produce a mature functional cytochrome	^27^
Q5VT66	Mitochondrial amidoxime reducing component 1	*MARC1/MTARC1*	337	Catalyzes the reduction of N-oxygenated molecules, acting as a counterpart of cytochrome P450 and flavin-containing monooxygenases in metabolic cycles	^28^
P17568	NADH dehydrogenase (ubiquinone) 1 β subcomplex, 7	*NDUFB7*	137	Accessory subunit of the mitochondrial NADH:ubiquinone oxidoreductase complex (Complex I)	^23^
O60936	Nucleolar protein 3	*NOL3*	208	Functions as an apoptosis repressor that blocks multiple modes of cell death	^29^
Q9HBL7	Plasminogen receptor (KT)	*PLGRKT*	147	Receptor for plasminogen	^30^

The characteristics of seven proteins found to be N-myristoylated by the experiments shown in Fig. 2 are summarized.

### DMAC1, HCCS, NDUFB7, and PLGRKT are N-myristoylated mitochondrial proteins

To assess the intracellular localization of the 7 proteins, an immunofluorescence analysis of COS-1 cells transfected with cDNA coding for the C-terminally FLAG-tagged protein was performed. In this analysis, MitoTracker Red and Hoechst 33342 were used as organelle markers for mitochondria and the nucleus, respectively. As shown in Fig. 2B, left panel and Supplementary Fig. S3, specific localization to mitochondria was observed with DMAC1, HCCS, NDUFB7, and PLGRKT, similar to MIC19, a known N-myristoylated mitochondrial protein. In contrast, selective localization to mitochondria was not observed with CLN3, MARC1, or NOL3. A line profile analysis of the images obtained further confirmed the specific localization of DMAC1, HCCS, NDUFB7, and PLGRKT to mitochondria (Fig. 2B, right panel).

### Protein N-myristoylation plays a critical role in the mitochondrial targeting of NDUFB7

**Figure 3:**
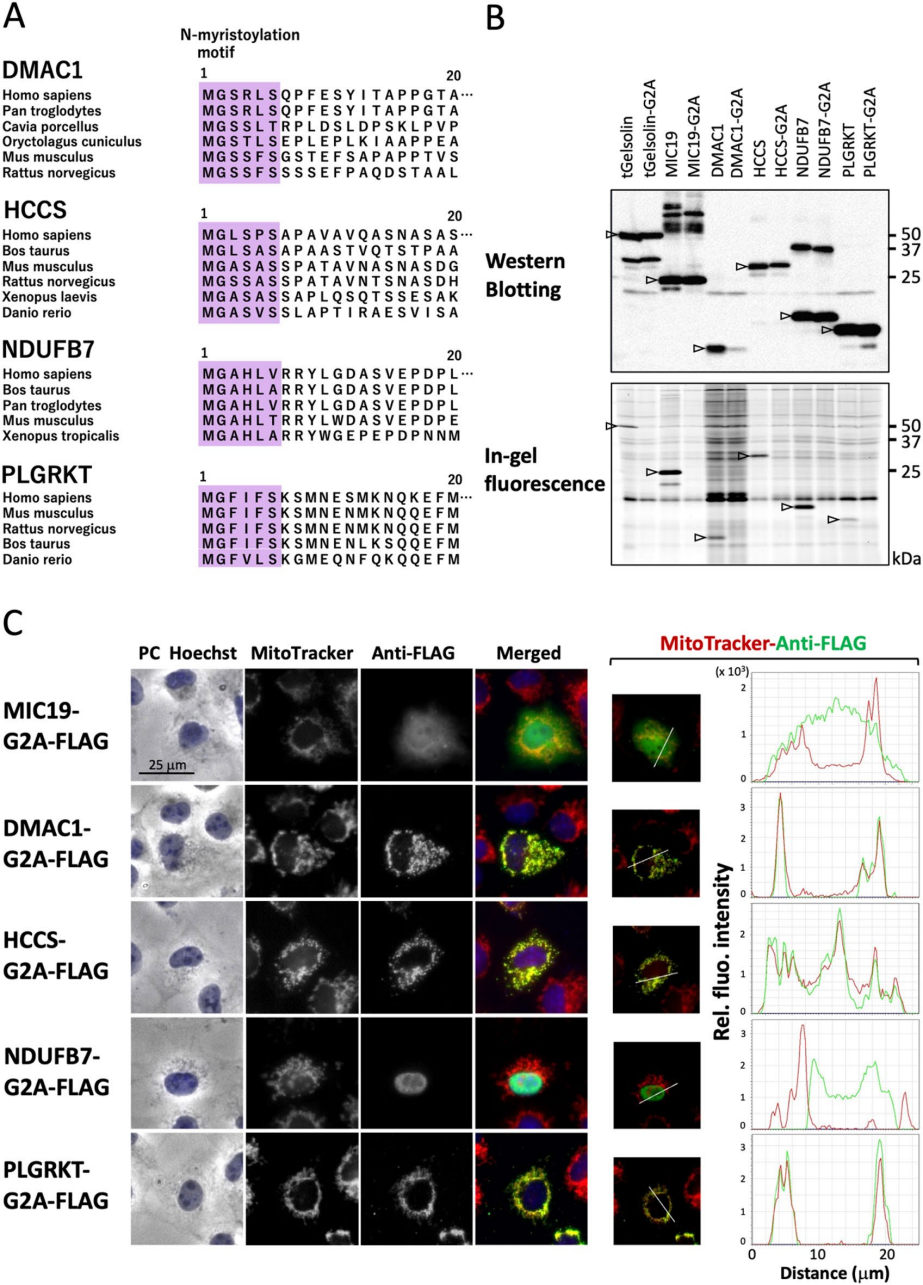
Protein N-myristoylation plays a critical role in the mitochondrial localization of NDUFB7. To clarify the role of protein N-myristoylation in the mitochondrial localization of DMAC1, HCCS, NDUFB7, and PLGRKT, non-myristoylatable G2A mutants were generated and their susceptibility to protein N-myristoylation and intracellular localization were evaluated. (**A**) Interspecies alignment of the N-terminal sequences of DMAC1, HCCS, NDUFB7, and PLGRKT. N-myristoylation motifs are shown in purple in N-terminal sequences. (**B**) Analysis of protein N-myristoylation of the WT and non-myristoylatable G2A mutants of DMAC1, HCCS, NDUFB7, and PLGRKT. cDNAs coding the WT and non-myristoylatable G2A mutants of four proteins were transfected into COS-1 cells. The expression of proteins was evaluated by Western blotting using an anti-FLAG antibody (upper panel). Protein N-myristoylation was evaluated by metabolic labeling with a myristic acid analog followed by click chemistry, as described in the Methods (lower panel). Arrows indicate the position of the expressed proteins. The images obtained by Western blotting or metabolic labeling were cropped. Uncropped full-length raw image data are shown in Supplementary Figure S4. (**C**) Analysis of the intracellular localization of the non-myristoylatable G2A mutant of DMAC1, HCCS, NDUFB7, and PLGRKT. The intracellular localization of the G2A mutant of the four proteins was assessed by an immunofluorescence analysis of COS-1 cells transfected with cDNA coding the C-terminally FLAG-tagged G2A mutant using an anti-FLAG antibody. MIC19-G2A-FLAG was used as a control non-myristoylatable G2A mutant. Hoechst and MitoTracker Red were used as organelle markers for the nucleus and mitochondria, respectively. Experiments were repeated 3 times and similar results were obtained. Representative data are shown. Abbreviation used: PC, phase contrast image. The results of the line profile analysis are shown. In the line profile analysis, corresponding line-scan graphs of the relative fluorescence intensities of green anti-FLAG fluorescence (green line) and red MitoTracker red fluorescence (red line) along the white line indicated in the merged images are shown.

As shown in Fig. 3A, the high conservation of the N-myristoylation motif was observed among vertebrates by the interspecies alignment of the N-terminal sequences of DMAC1, HCCS, NDUFB7, and PLGRKT.

To clarify the role of protein N-myristoylation in the mitochondrial localization of these four proteins, non-myristoylatable G2A mutants were generated and their susceptibility to protein N-myristoylation and intracellular localization were evaluated. As shown in Fig. 3B upper panel, the efficient expression of proteins was observed in the wild types and G2A mutants of four C-terminally FLAG-tagged proteins, except for the G2A mutant of DMAC1-FLAG. Metabolic labeling experiments revealed the efficient incorporation of Alk-Myr in wild-type proteins, whereas it was abolished in the G2A mutant (Fig. 3B, lower panel). When the intracellular localization of the G2A mutants of these four proteins was evaluated by an immunofluorescence analysis using an anti-FLAG antibody, marked differences were observed among the four proteins, as shown in Fig. 3C, left panel and Supplementary Fig. S5. Specific localization to mitochondria was observed for the G2A mutants of DMAC1, HCCS, and PLGRKT, similar to the wild-type proteins. The G2A mutant of NDUFB7 localized exclusively to the nucleus (Fig. 3C, left panel, Supplementary Fig. S5, and Supplementary Fig. S7A, upper panel). These results were further confirmed by the line profile analysis of the images obtained, as shown in Fig. 3C, right panel, and Supplementary Fig. S7A, lower panel.

However, in the case of MIC19, in which protein N-myristoylation was shown to be required for mitochondrial targeting, the G2A mutant localized to the cytoplasm (Fig. 3C, left panel, Supplementary Fig. S5, and Supplementary Fig. S8B, upper panel). This result was further confirmed by the line profile analysis of the images obtained, as shown in Fig. 3C, right panel. Therefore, protein N-myristoylation occurring on NDUFB7 played a critical role in mitochondrial localization, whereas that of DMAC1, HCCS, and PLGRKT did not affect the mitochondrial localization of these proteins.

### Structure of NDUFB7

**Figure 4:**
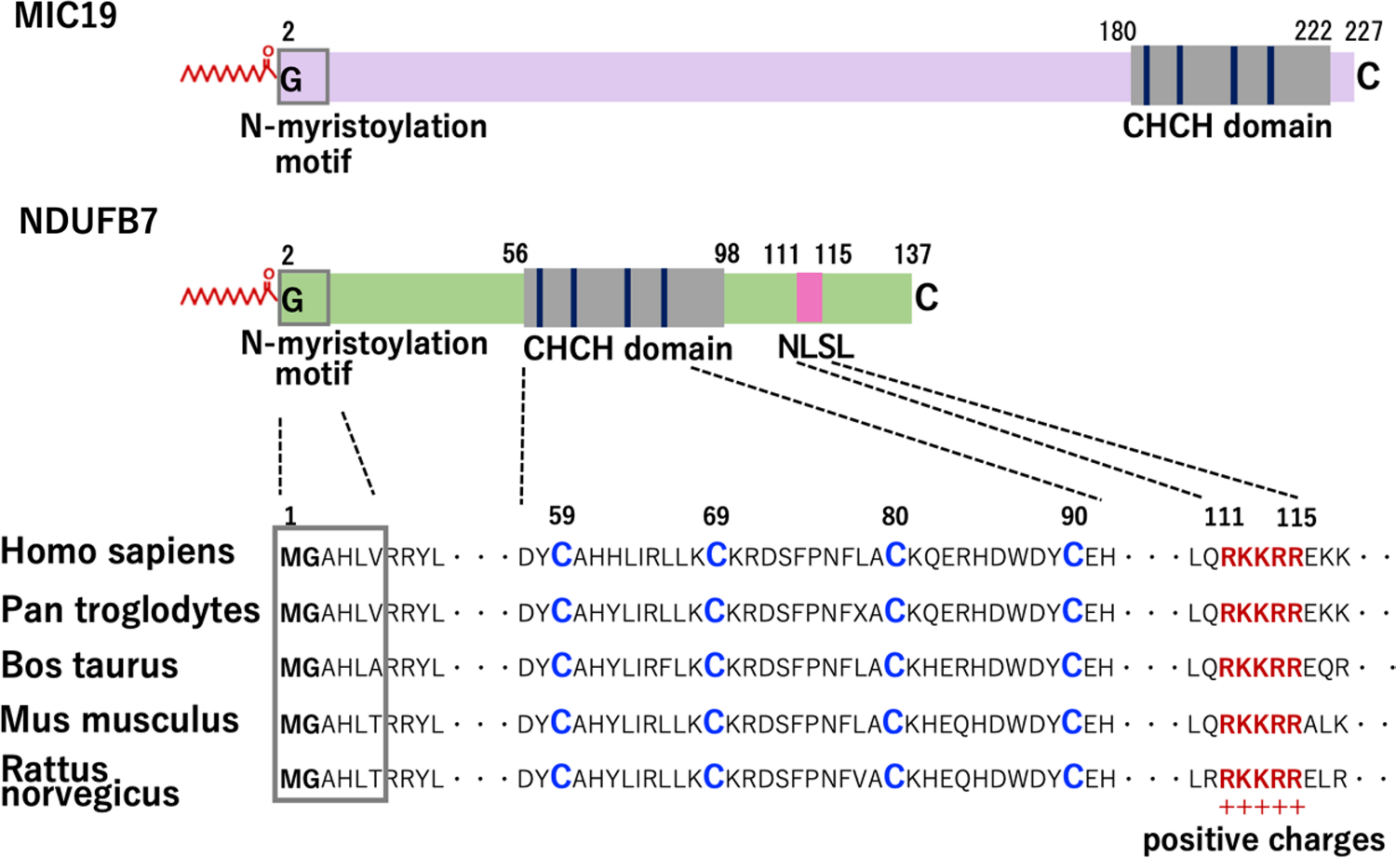
Structure of NDUFB7. Schematic representation of the structures of MIC19 and NDUFB7. Interspecies alignments of the N-myristoylation motif, CHCH domain, and nuclear localization signal-like (NLSL) sequence of NDUFB7 are shown. N-myristoylation motifs were boxed. Four conserved Cys residues in the CHCH domain were indicated by the blue characters in the amino acid sequences. Conserved RKKRR sequences in the NLSL sequence were indicated by the red characters in the amino acid sequences.

Similar to MIC19, NDUFB7 is a member of the CHCHD-containing protein family^31^. The structures of NDUFB7 and MIC19 are shown in Fig. 4.

In addition to the N-terminal N-myristoylation motif, NDUFB7 contains CHCHD at positions 56 to 98. CHCHD contains two Cx9C motifs at positions 59 to 69 and 80 to 90 that are predicted to form a helix-coil-helix structure, permitting the formation of intramolecular disulfide bonds^32^. Since the non-myristoylatable G2A mutant of NDUFB7 specifically localized to the nucleus, NDUFB7 was predicted to contain a nuclear localization signal-like sequence (NLSL). When the two public WWW server-based prediction programs for nuclear localization signals, cNLS Mapper (http://nls-mapper.iab.keio.ac.jp/cgi-bin/NLS_Mapper_form.cgi)^33^ and NucPred (http://www.sbc.su.se/~maccallr/nucpred)^34^, were applied to the amino acid sequence of NDUFB7, potential nuclear localization signals were predicted at positions 109 to 118 (LQRKKRREKK) and 111 to 115 (RKKRR), respectively. As shown in Fig. 4, the high conservation of the N-myristoylation motif, CHCHD, and NLSL was observed among vertebrates by the interspecies alignment of the protein sequence of NDUFB7.

### Positive charge cluster localized to the C-terminal region functions as a nuclear localization signal of the non-myristoylatable G2A mutant of NDUFB7

**Figure 5:**
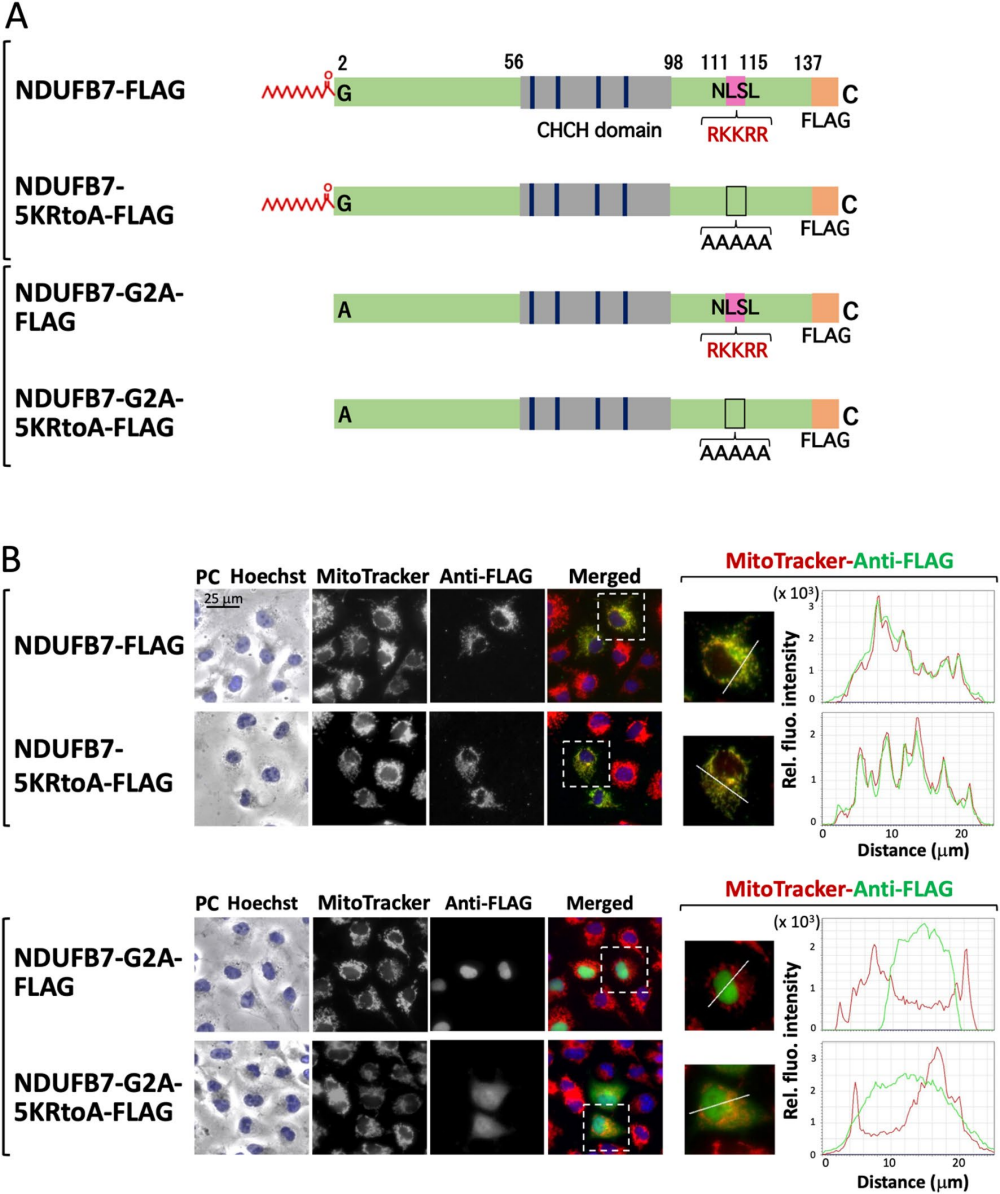
Positive charge cluster localized in the C-terminal region functions as a nuclear localization signal of the non-myristoylatable G2A mutant of NDUFB7. To establish whether the positive charge cluster at positions 111 to 115 of NDUFB7 function as a nuclear localization signal, two mutants in which amino acids at this positions of NDUFB7-FLAG and NDUFB7-G2A-FLAG were changed to Ala (NDUFB7-5KRtoA-FLAG and NDUFB7-G2A-5KRtoA-FLAG, respectively) were generated (Fig. 5A), and their susceptibility to protein N-myristoylation and their intracellular localization were evaluated. The results obtained on protein expression and protein N-myristoylation from a Western blotting analysis and metabolic labeling experiments, respectively, are shown in Supplementary Figure S6. (**A**) Structure of NDUFB7 mutants lacking the C-terminal positive charge cluster. (**B**) Analysis of the intracellular localization of NDUFB7 mutants lacking the C-terminal positive charge cluster. The intracellular localization of NDUFB7-FLAG, NDUFB7-5KRtoA-FLAG, NDUFB7-G2A-FLAG, and NDUFB7-G2A-5KRtoA-FLAG was assessed by an immunofluorescence analysis of COS-1 cells transfected with cDNA coding these four proteins using an anti-FLAG antibody. Hoechst and MitoTracker Red were used as organelle markers for the nucleus and mitochondria, respectively. Experiments were repeated 3 times and similar results were obtained. Representative data are shown. Abbreviation used: PC, phase contrast image. The results of the line profile analysis are shown. In the line profile analysis, corresponding line-scan graphs of the relative fluorescence intensities of green anti-FLAG fluorescence (green line) and red MitoTracker red fluorescence (red line) along the white line indicated in the merged images are shown.

Two public WWW server-based prediction programs for nuclear localization signals predicted a possible nuclear localization signal and the predicted nuclear localization signal overlapped at positions 111 to 115 (RKKRR). Therefore, we attempted to clarify whether the positive charge cluster at this position functioned as a nuclear localization signal. Two mutants in which amino acids at positions 111 to 115 of NDUFB7-FLAG and NDUFB7-G2A-FLAG were changed to Ala (NDUFB7-5KRtoA-FLAG and NDUFB7-G2A-5KRtoA-FLAG, respectively) were generated (Fig. 5A) and their susceptibility to protein N-myristoylation and their intracellular localization were evaluated.

As shown in Supplementary Fig. S6, efficient protein expression and protein N-myristoylation were equally observed on NDUFB7-FLAG and NDUFB7-5KRtoA-FLAG. In contrast, the level of protein expression of NDUFB7-G2A-5KRtoA-FLAG was lower than that of NDUFB7-G2A-FLAG. As expected, protein N-myristoylation was not observed on these two G2A mutants. As shown in Fig. 5B upper left panel, NDUFB7-5KRtoA-FLAG specifically localized to mitochondria, similar to wild-type NDUFB7-FLAG. In contrast, NDUFB7-G2A-5KRtoA-FLAG localized to the cytoplasm, whereas NDUFB7-G2A-FLAG specifically localized to the nucleus (Fig. 5B lower left panel and Supplementary Fig. S7, A, B upper panels). These results were further confirmed by the line profile analysis of the images obtained, as shown in Fig. 5B, right panels and Supplementary Fig. S7, A B, lower panels.

### CHCHD plays a critical role in the mitochondrial localization of NDUFB7

**Figure 6:**
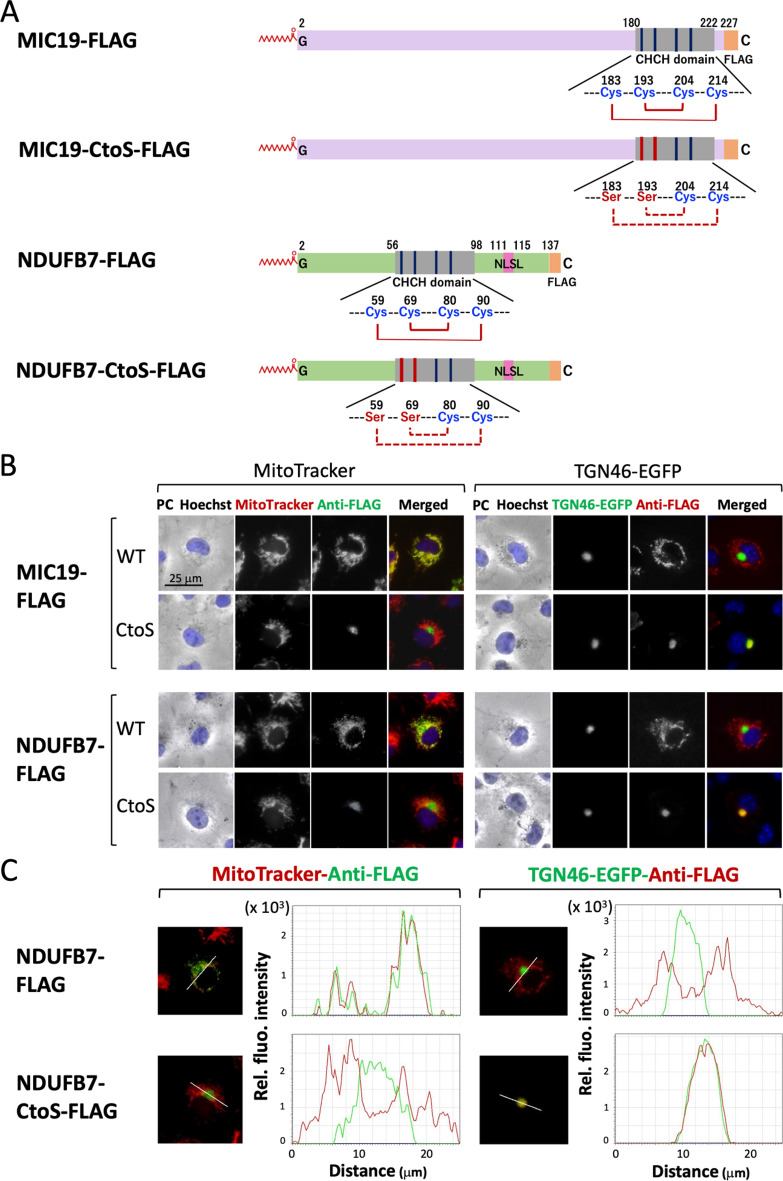
The CHCH domain plays a critical role in the mitochondrial localization of NDUFB7. To elucidate the role of the CHCH domain in NDUFB7 in the mitochondrial localization of this protein, two mutants in which Cys residues at positions 59 and 69 in the wild type and G2A mutant of NDUFB7 were changed to Ser (NDUFB7-CtoS-FLAG and NDUFB7-G2A-CtoS-FLAG, respectively) were generated (Fig. 6A and Supplementary Fig. S8A) and their susceptibility to protein N-myristoylation and their intracellular localization were evaluated. As controls for this analysis, two MIC19 mutants in which the corresponding Cys residues at 183 and 193 in the wild type and G2A mutant of MIC19-FLAG were changed to Ser (MIC19-CtoS-FLAG and MIC19-G2A-CtoS-FLAG, respectively) were generated and the same experiments were performed. The results obtained on protein expression and protein N-myristoylation from a Western blotting analysis and metabolic labeling experiments, respectively, are shown in Supplementary Figure S9. (**A**) Structures of MIC19-FLAG, MIC19-CtoS-FLAG, NDUFB7-FLAG, and NDUFB7-CtoS-FLAG. (**B**) Analysis of the intracellular localization of MIC19-FLAG, MIC19-CtoS-FLAG, NDUFB7-FLAG, and NDUFB7-CtoS-FLAG. The intracellular localization of MIC19-FLAG, MIC19-CtoS-FLAG, NDUFB7-FLAG, and NDUFB7-CtoS-FLAG was assessed by an immunofluorescence analysis of COS-1 cells transfected with cDNA coding these four proteins using an anti-FLAG antibody. Hoechst, MitoTracker Red, and TGN-46-EGFP were used as organelle markers for the nucleus, mitochondria, and TGN, respectively. Experiments were repeated 3 times and similar results were obtained. Representative data are shown. Abbreviation used: PC, phase contrast image. (**C**) A line profile analysis of the intracellular localization of NDUFB7-FLAG and NDUFB7-CtoS-FLAG. The results of a line profile analysis are shown. In the line profile analysis, corresponding line-scan graphs of the relative fluorescence intensities of green anti-FLAG or TGN46-EGFP fluorescence (green line) and red MitoTracker red or anti-FLAG fluorescence (red line) along the white line indicated in the merged images are shown.

The Mia40/CHCHD4 import pathway has been shown to mediate the import of (CX_9_C)_2_ motif-carrying substrates into the intermembrane space (IMS) through outer membrane-localized TOM translocase^35^. The formation of two disulfide bonds among the (CX_9_C)_2_ motif in the substrates catalyzed by the oxidase Mia40/CHCHD4 plays a critical role in this transport pathway^31^. Regarding NDUFB7, two intra-molecular disulfide bridges between two Cys pairs (Cys59/Cys90 and Cys69/Cys80) were proposed to be formed by this system^32^. Therefore, we attempted to elucidate the role of CHCHD in NDUFB7 in the mitochondrial localization of this protein. To achieve this, two mutants in which Cys residues at positions 59 and 69 in the wild type and G2A mutant of NDUFB7 were changed to Ser (NDUFB7-CtoS-FLAG and NDUFB7-G2A-CtoS-FLAG, respectively) were generated (Fig. 6A and Supplementary Fig. S8A) and their susceptibility to protein N-myristoylation and their intracellular localization were evaluated.

As controls for this analysis, two MIC19 mutants in which the corresponding Cys residues at positions 183 and 193 in the wild type and G2A mutant of MIC19-FLAG were changed to Ser (MIC19-CtoS-FLAG and MIC19-G2A-CtoS-FLAG, respectively) were generated. As shown in Supplementary Fig. S9, efficient protein expression and effective protein N-myristoylation were equally observed on NDUFB7-FLAG and NDUFB7-CtoS-FLAG. In contrast, the protein expression level of NDUFB7-G2A-CtoS-FLAG was significantly lower than that of NDUFB7-G2A-FLAG. Protein N-myristoylation was not observed on NDUFB7-G2A-FLAG as expected. As shown in Fig. 6B, the CtoS mutant of NDUFB7-FLAG (NDUFG7-CtoS-FLAG) did not localize to mitochondria, but specifically localized to the Golgi apparatus (TGN), as was the case with MIC19-CtoS-FLAG. These results were further confirmed by the line profile analysis of the images obtained, as shown in Fig. 6C. In contrast, the CtoS mutant derived from the G2A mutant of NDUFB7 (NDUFB7-G2A-CtoS-FLAG) localized to the nucleus, as was the case with NDUFB7-G2A-FLAG (Supplementary Fig. S8B). Regarding the CtoS mutant of MIC19-G2A-FLAG (MIC19-G2A-CtoS-FLAG), a cytosolic distribution was observed, similar to MIC19-G2A-FLAG, as shown in Supplementary Fig. S8B upper panels. These results indicated that CHCHD played a critical role in the mitochondrial localization of NDUFB7, as was the case for MIC19. Furthermore, protein N-myristoylation and CHCHD were both required for the proper mitochondrial targeting and localization of NDUFB7.

## Discussion

In an attempt to identify novel human N-myristoylated proteins from human cDNA clones in human cDNA resources, we previously found that two mitochondrial outer membrane proteins, SAMM50 and TOMM40, were N-myristoylated integral membrane proteins^18^. The protein N-myristoylation of SAMM50, but not TOMM40, was also shown to be required for mitochondrial targeting. In the present study, we searched for human N-myristoylated mitochondrial proteins in which protein N-myristoylation was required for their specific localization to mitochondria. Among the 1,705 human genes listed in MitoProteome, 12 candidate genes for N-myristoylated mitochondrial proteins were selected and their susceptibility to protein N-myristoylation and their intracellular localization were evaluated, as previously described^14–18,36^. As a result, 7 proteins (CLN3, DMAC1, HCCS, MARC1, NDUFB7, NOL3, and PLGRKT) were found to be N-myristoylated proteins. The endogenous protein N-myristoylation occurring on these 7 proteins expressed in HeLa cells have been reported in recent reports ^37,38^.

Among them, four proteins, DMAC1, HCCS, NDUFB7, and PLGRKT, were identified as N-myristoylated proteins that specifically localized to mitochondria (Fig. 2). None of these proteins contain predicted cleavable presequences that direct mitochondrial localization. Among these proteins, DMAC1, HCCS, and NDUFB7 have been shown to be mitochondrial proteins. DMAC1 and NDUFB7 were found to play important roles in the assembly of complex I of the mitochondrial respiratory chain as an assembly factor and an accessory subunit, respectively^23^. Both DMAC1 and NDUFB7 localize to the inner membrane and associate with complex I of the mitochondrial respiratory chain^23,39^. The import pathway for NDUFB7 has been characterized. NDUFB7 was first synthesized by cytosolic ribosomes in the cytoplasm, and imported into intermembrane space through outer membrane-localized TOM complex in an unfolded reduced form, then folded and oxidized by the disulfide relay-dependent Mia40/CHCHD4 import machinery^39,40^. The import pathway for DMAC1 has not been characterized so far.

HCCS localizes to the intermembrane space and functions as a lyase that catalyzes the covalent linking of the heme group to the cytochrome C apoprotein to produce mature functional cytochrome C^27^.

The localization and import pathway of HCCS has been well characterized in yeast^41^. HCCS was synthesized in the cytosol and directly imported to intermembrane space through outer membrane-localized TOM complex, then localized to the outer surface of the inner membrane.

Different from DMAC1, NDUFB7 and HCCS, PLGRKT has been shown to be a cell surface receptor for plasminogen that regulates cell surface plasminogen activation^30^. The presence and biological function of PLGRKT in mitochondria have not yet been reported. Thus, intramitochondrial localization and import pathway for PLGRKT have not been characterized yet.

An analysis of the intracellular localization of non-myristoylatable G2A mutants indicated that protein N-myristoylation occurring on NDUFB7, an accessory subunit of human mitochondrial respiratory chain complex I, played a vital role in the mitochondrial localization of this protein (Fig. 3). NDUFB7 is a member of the CHCHD-containing family of proteins (CHCHD proteins), which are imported into the mitochondrial IMS, and it has been proposed to stabilize assembled mitochondrial respiratory chain complex I by binding to its surface^32^. Mitochondrial respiratory chain complex I is the largest complex in the oxidative phosphorylation system. It comprises 14 central subunits, 31 accessory subunits, and at least 15 assembly factors^40^. Significant advances have recently been made in elucidating the structure and assembly steps of complex I. NDUFB7 is an accessory subunit of module P_D_-b. A critical role has been suggested for NDUFB7 in the assembly of complex I in the yeast *Yarrowia lipolytica*^42^. Furthermore, from in vitro experiments, the absence of any of the accessory subunits of the P_D_-b module may critically affect the assembly and function of human complex I^23,42,43^.

In fully assembled complex I, the module P_D_-b is composed of the central subunit ND5 and accessory subunits NDUFB2, NDUFB3, NDUFB7, NDUFB8, and NDUFB9^40,43^. Deleterious changes in proteins of the P_D_-b module have been described and associated with complex I disorders^44–46^. Regarding NDUFB7, a recent study reported that an intronic mutation leading to a cryptic splice site in the NDUFB7 gene caused a mitochondrial disorder^47^. In this case, an intronic mutation in NDUFB7 caused severe congenital lactic acidosis and hypertrophic cardiomyopathy. The detected mutation resulted in a significant reduction in the NDUFB7 protein and reduced complex I activity. It was also revealed that complementation studies with the expression of wild-type NDUFB7 in patient fibroblasts normalized complex I function. Therefore, NDUFB7 plays an important role in the formation and function of complex I.

The CHCHD-containing family of proteins are proteins that localize to the mitochondrial IMS and contain (CX_3_C)_2_ or (CX_9_C)_2_ motifs^31^. These CHCHD proteins are specifically transported into mitochondria by the disulfide relay-dependent Mia40/CHCHD4 import machinery^35^. They are involved in mitochondrial biogenesis, bioenergetics, dynamics, and quality control in the IMS^31^. Similar to other CHCHD proteins, CHCHD is required for the mitochondrial localization of NDUFB7; however, the present study revealed that NDUFB7 required protein N-myristoylation in addition to CHCHD. G2A mutant in which protein N-myristoylation was inhibited did not localize to mitochondria, but specifically localized to the nucleus (Figs. 3, 5). The nuclear localization of the G2A mutant of NDUFB7 was directed by the NLSL RKKRR, located at positions 111–115 (Fig. 5). This result indicated that protein N-myristoylation-mediated mitochondrial localization is preferential over nuclear localization mediated by the NLSL RKKRR.

A known CHCHD protein that requires protein N-myristoylation for mitochondrial localization is MIC19 (CHCHD3), an N-myristoylated mitochondrial inner membrane-binding protein that interacts with the luminal surface of the MICOS complex and is involved in the formation of cristae^18,48^. In addition to MIC19, protein N-myristoylation has been proposed to occur on another CHCHD protein, MIC25 (CHCHD6), which localizes to the mitochondrial IMS. A phylogenic analysis indicated that human MIC19 and MIC25 resulted from a gene duplication at the root of vertebrates^49^. Therefore, they are both orthologous to MIC19 in yeast and likely the result of whole genome duplication^50^. Regarding the mitochondrial localization of MIC19 and MIC25, neither a canonical cleavable N-terminal mitochondrial targeting signal (presequence) nor another specific mitochondrial targeting signal has been identified. However, as for MIC19, protein N-myristoylation and CHCHD were both essential for the proper mitochondrial localization of this protein^48^.

MIC25 was predicted to be *N*-myristoylated; however, direct biochemical evidence for protein N-myristoylation has not yet been reported. We previously showed that exogenously and endogenously expressed MIC19 and MIC25 were efficiently *N*-myristoylated^18^. Overexpressed MIC19 specifically localized to mitochondria, whereas the non-myristoylatable G2A mutant of MIC19 localized to the cytoplasm, confirming the requirement of protein N-myristoylation for the mitochondrial localization of MIC19. However, the majority of overexpressed N-myristoylated MIC25 was detected in the cytoplasm, but not in mitochondria. A similar cytosolic localization was observed for the non-myristoylatable G2A mutant of MIC25. Therefore, protein N-myristoylation did not appear to play a critical role in the mitochondrial localization of MIC25. The mechanisms responsible for differences in the role of protein N-myristoylation in the mitochondrial localization of MIC19 and MIC25 remains unclear.

A mechanism for the protein N-myristoylation-dependent mitochondrial localization of Mic19 was recently proposed using yeast Mic19^51^. This study demonstrated that the CHCHD-mediated mitochondrial translocation of Mic19 was inhibited by an unfolded domain in its N-terminal region, called the DAF domain. Protein N-myristoylation circumvented this inhibitory effect by mediating the binding of MIC19 to Tom20 in the TOM complex and to the mitochondrial outer membrane, thereby achieving the efficient mitochondrial localization of MIC19. It remains unclear whether protein N-myristoylation plays a similar role with respect to NDUFB7. However, since there is no DUF domain-like unfolded domain in the N-terminal region of NDUFB7, it is unlikely that the protein N-myristoylation of NDUFB7 circumvented the inhibitory effects of the DUF domain-like unfolded domain on mitochondrial localization. In addition, the non-myristoylatable G2A mutant of NDUFB7 was shown to localize to the nucleus rather than to the cytoplasm, different from the case for MIC19 (Fig. 5B lower panel, Supplementary Fig. S5, Supplementary Fig. S7A). Furthermore, the nuclear localization of the non-myristoylatable G2A mutant of NDUFB7 was mediated by the NLSL RKKRR, located at positions 111 ~ 115 (Fig. 5B lower panel, Supplementary Fig. S7). Therefore, protein N-myristoylation in NDUFB7 appears to function in the inhibition of nuclear localization mediated by the NLSL RKKRR rather than circumventing the suppressive effects of the DUF domain on mitochondrial localization, as observed for Mic19. It currently remains unclear why a positive charge amino acid cluster, such as RKKRR, is present in NDUFB7. In some proteins, positively charged amino acids play a role in protein–protein interactions and the assembly of protein complexes. For example, electrostatic interactions between positively and negatively charged residues play critical roles in the structure and function of mammalian respiratory complex I^39,52^. Therefore, the amino acid sequence RKKRR in NDUFB7 may be involved in the function of NDUFB7 as an accessory subunit of respiratory chain complex I.

In MIC19, protein N-myristoylation played a critical role not only in mitochondrial localization, but also in interactions with SAMM50 localized to the mitochondrial outer membrane^48^. In the case of NDUFB7, protein N-myristoylation was first reported on bovine NDUFB7, and this modification was proposed to function as a membrane anchor to the mitochondrial inner membrane^53^. Concerning the protein–protein interactions mediated by protein N-myristoylation of NDUFB7, recent study on the structure of mammalian mitochondrial complex I indicated that the N-terminal myristoyl group of NDUFB7 is bound in a groove on ND5 formed by TMH12ND5, TMH13ND5 and TMH15ND5^39^. Thus, it is quite probable that the protein–protein interactions mediated by protein N-myristoylation are involved in the function of NDUFB7 as an accessory subunit of respiratory chain complex I.

**Figure 7:**
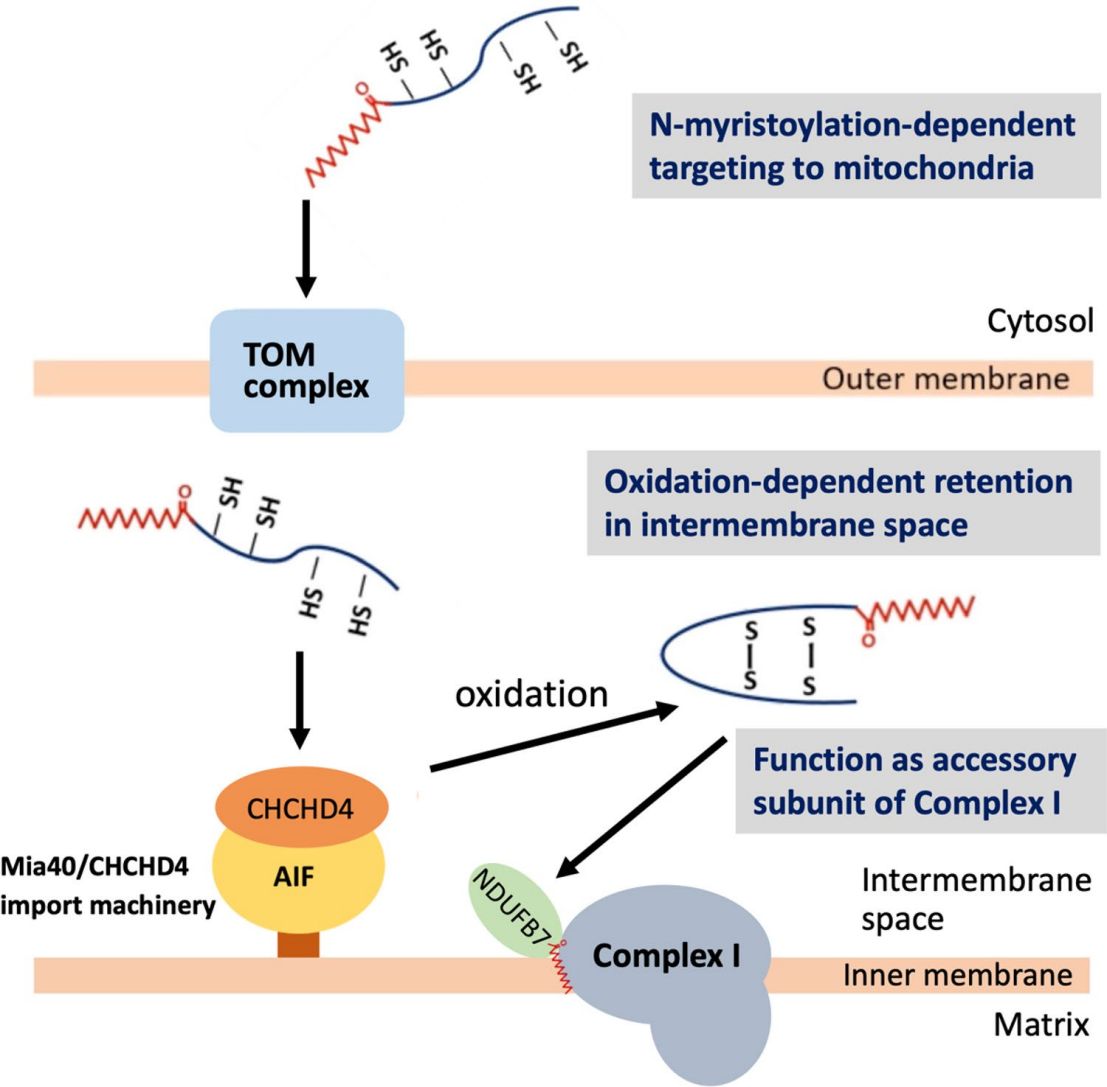
Schematic representation of possible roles of protein N-myristoylation and the CHCH domain in the mitochondrial targeting and localization of NDUFB7. NDUFB7 was synthesized by cytosolic ribosomes in the cytoplasm, targeted to the mitochondrial outer membrane, and then translocated across the outer membrane through outer membrane-localized TOM translocase (TOM complex) by a protein N-myristoylation-dependent mechanism. The Mia40/CHCHD4 import machinery then mediated the oxidation-dependent retention of NDUFB7 in the intermembrane space. NDUFB7 functioned as an accessory subunit of complex I of the mitochondrial respiratory chain (Complex I). In this mechanism, protein N-myristoylation and CHCH-domain were both required for the proper mitochondrial localization of NDUFB7.

Schematic representation of possible roles of protein N-myristoylation and the CHCH domain in the mitochondrial targeting and localization of NDUFB7 is shown in Fig. 7.

NDUFB7 was first synthesized by cytosolic ribosomes in the cytoplasm, targeted to the mitochondrial outer membrane, and then translocated across the outer membrane through outer membrane-localized TOM translocase (TOM complex) by a protein N-myristoylation-dependent mechanism. The Mia40/CHCHD4 import machinery then mediated the oxidation-dependent retention of NDUFB7 in the intermembrane space. The NDUFB7 localized in the intermembrane space functioned as an accessory subunit of complex I of the mitochondrial respiratory chain (Complex I). In this mechanism, protein N-myristoylation and CHCH-domain were both required for the proper mitochondrial localization of NDUFB7. However, the relative roles of protein N-myristoylation and CHCH-domain in the intracellular localization and function of NDUFB7 remained to be elucidated. Further analyses are required to fully elucidate the role of protein N-myristoylation in the intracellular localization and function of NDUFB7.

## Methods

### Materials

cDNA coding human MIC19 was purchased from Promega Corp. (Madison, WI, USA). Other human cDNAs were obtained from the RIKEN BioResource Research Center (RIKEN BRC, Tsukuba, Japan) through the National Bio-Resource Project of the MEXT, Japan^54–57^. ECL prime Western blotting detection reagents were from GE Healthcare (Buckinghamshire, UK). The dye terminator cycle sequencing kit, Lipofectamine LTX and Plus reagents, MitoTracker Red CMXRos, and Hoechst 33342 were from Life Technologies Corporation (Carlsbad, CA, USA). An anti-FLAG monoclonal antibody and anti-mouse IgG-FITC antibody were from Sigma (St. Louis, MO, USA). An anti-mouse IgG-ALEXA594 antibody was from Thermo Scientific (Rockford, IL, USA). 13-Tetradecynoic acid (Alk-Myr) was from Cayman (Ann Arbor, MI, USA). Azide TAMRA (Az-TAMRA) was from Click Chemistry Tools (Scottsdale, AZ, USA). Tris(2-carboxyethyl)phosphine hydrochloride (TCEP) and tris[(1-benzyl-1*H*-1,2,3-triazol-4-yl)methyl]amine (TBTA) were from Sigma (St. Louis, MO, USA). Protein G-HRP conjugate was from Bio-Rad (Hercules, CA, USA). The other reagents used were from Wako Pure Chemical (Osaka, Japan) or Daiichi Pure Chemicals (Tokyo, Japan) and were of analytical or DNA grade.

### Prediction of protein N-myristoylation using prediction programs

Two public WWW server-based prediction programs for protein N-myristoylation, MYR Predictor (http://mendel.imp.ac.at/myristate/SUPLpredictor.htm)^24^ and Myristoylator (http://www.expasy.org/tools/myristoylator/)^25^, were used to predict protein N-myristoylation. The entire amino acid sequences deduced from the nucleotide sequences of the ORFs were used as the query.

### Plasmid construction

The nucleotide sequences of oligonucleotides used for plasmid construction are summarized in Supplementary Tables S3, S4, and S5. The plasmids pcDNA3-tGelsolin-FLAG, pcDNA3-FLAG, and pcDNA3-TGN46-EGFP were constructed as previously described^58,59^. pcDNA3 plasmids containing the cDNAs coding tGelsolin fusion proteins with the 10 N-terminal amino acids of the proteins listed in the MitoProteome protein database at the N terminus (pcDNA3-X(N10)-tGelsolin-FLAG) were constructed using the oligonucleotides listed in Table S3 and the plasmids pcDNA3-tGelsolin-FLAG and pcDNA3-FLAG, as described in Table S6. pcDNA3 plasmids, including cDNA coding C-terminally FLAG-tagged full-length proteins and their mutants, were constructed by PCR or site-directed mutagenesis, as summarized in Supplementary Tables S6 and S7.

### Transfection of cells

COS-1 (a simian virus 40-transformed African green monkey kidney cell line, American Type Culture Collection) cells were maintained in Dulbecco’s modified Eagle’s medium (DMEM; Gibco BRL [Palo Alto, CA, USA]) supplemented with 10% fetal calf serum (FCS; Gibco BRL). Cells (2 × 10^5^) were plated onto 35-mm dishes 1 day before transfection. pcDNA3 constructs (2 μg) containing cDNA coding FLAG-tagged or EGFP-fusion proteins were transfected into cells on each plate using 2.5 μL of Lipofectamine LTX and 2 μL of Plus reagent in 1 mL of serum-free medium^18^. After an incubation at 37 °C for 5 h, serum-containing medium was added and cells were incubated again at 37 °C for the appropriate periods.

### Metabolic labeling of cells

The metabolic labeling of cells with the myristic acid analog (Alk-Myr) was performed as previously described^18^. Cells (2 × 10^5^) were transfected with pcDNA3 constructs (2 μg) containing cDNA as described above and incubated at 37 °C for 12 h. They were then washed once with 1 mL of serum-free DMEM and incubated at 37 °C for 10 h in 1 mL of DMEM (+ 2% FCS) containing 25 μM Alk-Myr. Cells were then washed three times with Dulbecco’s phosphate-buffered saline (DPBS), harvested, and lysed with 200 μL of RIPA buffer (50 mM Tris–HCl (pH 7.5), 150 mM NaCl, 1% Nonidet P-40, 0.5% sodium deoxycholate, 0.1% SDS, and protease inhibitors) on ice for 20 min. Samples labeled with Alk-Myr were reacted with Az-TAMRA by click chemistry and protein N-myristoylation was analyzed by an in-gel fluorescence analysis^36^.

### Cu(I)-catalyzed azide-alkyne cycloaddition (CuAAC)

Cell lysates labeled with Alk-Myr (46 µL) were reacted with 4 µL of a freshly premixed click chemistry reaction cocktail (1 µL Az-TAMRA [5 mM], 1 µL TCEP [50 mM], 1 µL TBTA [5 mM], and 1 µL CuSO_4_·5H_2_O [50 mM]) in a total reaction volume of 50 µL at room temperature for 1 h. After CuAAC, 500 µL of MeOH was added to the samples and they were stored at − 80 °C overnight. After centrifugation at 15,000 rpm at 4 °C for 30 min, the supernatant was removed. Thereafter, the pellet was washed with 500 µL of MeOH and air dried^18^. Samples were denatured by sonication in SDS-sample buffer and then subjected to SDS-PAGE. An in-gel fluorescence analysis of SDS-PAGE gels was performed using Typhoon FLA9500 (GE Healthcare Bio-Sciences AB, Uppsala, Sweden)^60^.

### Western blotting

Proteins were resolved by 12.5% SDS-PAGE and then transferred to an Immobilon-P transfer membrane. After blocking with non-fat milk, the membrane was probed with a primary antibody, as previously described^61^. Immunoreactive proteins were specifically detected by an incubation with a protein G-HRP conjugate. The membrane was developed using ECL Prime Western blotting detection reagent and bands were detected using a MicroChemi Chemiluminescence Imaging System (Berthold Technologies, Bad Wildbad, Germany).

### Immunofluorescence analysis and fluorescence microscopy

An immunofluorescence analysis of transfected cells was performed 24 h after transfection^62^. After staining with Hoechst 33342 and MitoTracker Red, cells were washed with DPBS, fixed in 4% paraformaldehyde in DPBS for 15 min, and permeabilized with 0.1% Triton X-100 in DPBS for 10 min at room temperature, followed by washing with 0.1% gelatin in DPBS^36^. Permeabilized cells were incubated with a specific antibody in DPBS at room temperature for 1 h. After washing with 0.1% gelatin in DPBS, cells were incubated with the anti-IgG-FITC or anti-IgG-ALEXA594 antibody at room temperature for 1 h. After washing with 0.1% gelatin in DPBS, cells were observed using a Leica AF7000 fluorescence microscope (Leica, Solmser, Germany)^36^. A line profile analysis was performed using the Line Profile Tool of Leica LAS AF software (Leica, Solmser, Germany). In this analysis, white lines in merged panels were converted to line profiles using the Line Profile Tool.

### Supplementary Information

Supplementary Information.

## Data Availability

All data generated or analyzed during this study are included in this published article and its supplementary information files.
