# Supplementary material for: Protein N-myristoylation plays a critical role in the mitochondrial localization of human mitochondrial complex I accessory subunit NDUFB7
Source: Sci Rep. 2023 Dec 27;13:22991. doi: 10.1038/s41598-023-50390-z (PMC10752898; doi:10.1038/s41598-023-50390-z)
Supplement: Supplementary file 1 — Supplementary Information. [file 41598_2023_50390_MOESM1_ESM.docx]

**Protein N-myristoylation plays a critical role in the mitochondrial localization of human mitochondrial complex I accessory subunit NDUFB7**

Haruna Harada^1^, Koko Moriya^1^, Hirotsugu Kobuchi^4^, Naotada Ishihara^3^, & Toshihiko Utsumi^1, 2^*

^1^ Graduate School of Sciences and Technology for Innovation, Yamaguchi University, Yamaguchi, Japan

^2^ Department of Biological Chemistry, Faculty of Agriculture, Yamaguchi University, Yamaguchi, Japan

^3^ Department of Biological Sciences, Graduate School of Science, Osaka University, Osaka, Japan

^4^ Department of Cell Chemistry, Okayama University Graduate School of Medicine, Dentistry and Pharmaceutical Sciences, Okayama, Japan

*Corresponding author

E-mail: utsumi@yamaguchi-u.ac.jp

**Supplementary Figure S1.**

Raw images for Fig.1C.

Lanes marked X have been cropped from the blots shown in Fig. 1C.

**Supplementary Figure S2**

Raw images for Fig.2A.

**Supplementary Figure S3**

**Analysis of the intracellular localization of CLN3, DMAC1, HCCS, MARC1, NDUFB7, NOL3, and PLGRKT by an immunofluorescence microscopic analysis.**

The intracellular localization of seven proteins was assessed by an immunofluorescence analysis of COS-1 cells transfected with cDNA coding C-terminally FLAG-tagged full-length proteins using an anti-FLAG antibody. The results of the same experiments presented in Fig. 2B are shown. Images including more than two transfected cells in a wider area are shown. Experiments were repeated 3 times and similar results were obtained. Representative data are shown. Abbreviation used: PC, phase contrast image.

**Supplementary Figure S4**

Raw images for Fig.3B.

**Supplementary Figure S5**

**Analysis of the intracellular localization of non-myristoylatable G2A mutants of DMAC1, HCCS, NDUFB7, and PLGRKT.**

The intracellular localization of the G2A mutants of four proteins was assessed by an immunofluorescence analysis of COS-1 cells transfected with cDNA coding C-terminally FLAG-tagged G2A mutants using an anti-FLAG antibody. The results of the same experiments presented in Fig. 3C are shown. Images including more than two transfected cells in a wider area were shown. Experiments were repeated 3 times and similar results were obtained. Representative data are shown. Abbreviation used: PC, phase contrast image.

**Supplementary Figure S6**

A. Analysis of the protein N-myristoylation of NDUFB7-FLAG, NDUFB7-5KRtoA-FLAG, NDUFB7-G2A-FLAG, and NDUFB7-G2A-5KRtoA-FLAG expressed in transfected COS-1 cells by metabolic labeling.

cDNAs encoding C-terminally FLAG-tagged proteins were transfected into COS-1 cells and cells were labeled with a myristic acid analog. The expression of proteins was evaluated by Western blotting using an anti-FLAG antibody (left panel). Protein N-myristoylation was evaluated by metabolic labeling followed by click chemistry, as described in the Methods (right panel).

B. Raw images for Supplementary Fig. S6A.

**Supplementary Figure S7**

**Positive charge cluster localized in the C-terminal region functions as a nuclear localization signal of the non-myristoylatable G2A mutant of NDUFB7**

To assess whether the positive charge cluster at positions 111 to 115 of NDUFB7 functions as a nuclear localization signal, the intracellular localization of NDUFB7-G2A-5KRtoA-FLAG was compared with that of NDUFB7-G2A-FLAG.

**A: Analysis of the intracellular localization of NDUFB7-G2A-FLAG.**

The intracellular localization of NDUFB7-G2A-FLAG was assessed by an immunofluorescence analysis using an anti-FLAG antibody. Hoechst and MitoTracker Red were used as organelle markers for the nucleus and mitochondria, respectively. Experiments were repeated 3 times and similar results were obtained. Representative data are shown. Abbreviation used: PC, phase contrast image.

The results of a line profile analysis are shown. A close-up image of the area surrounded by a white dotted box in the merged image was used for the analysis. In the line profile analysis, corresponding line-scan graphs of the relative fluorescence intensities of green anti-FLAG fluorescence (green line) and red MitoTracker red fluorescence (red line) (lower left panels) or blue Hoechst fluorescence (blue line) (lower right panels) along the white line indicated in the merged image are shown.

**B: Analysis of the intracellular localization of NDUFB7-G2A-5KRtoA-FLAG.**

The intracellular localization of NDUFB7-G2A-5KRtoA-FLAG was assessed by the same methods as those described in A. Experiments were repeated 3 times and similar results were obtained. Representative data are shown. The results of a line profile analysis of a close-up image of the area surrounded by a white dotted box in the merged image are shown.

**Supplementary Figure S8**

**Protein N-myristoylation is required for the CHCH domain-dependent mitochondrial localization of NDUFB7**

To elucidate the role of protein N-myristoylation in the CHCH domain-dependent mitochondrial localization of MIC19 and NDUFB7, the intracellular localization of the non-myristoylatable mutants of MIC19-CtoS-FLAG and NDUFB7-CtoS-FLAG (MIC19-G2A-CtoS-FLAG and NDUFB7-G2A-CtoS-FLAG, respectively) were compared with those of MIC19-G2A-FLAG and NDUFB7-G2A-FLAG.

**A: Structure of MIC19-G2A-FLAG, MIC19-G2A-CtoS-FLAG, NDUFB7-G2A-FLAG, and NDUFB7-G2A-CtoS-FLAG**

**B: Analysis of the intracellular localization of MIC19-G2A-FLAG, MIC19-G2A-CtoS-FLAG, NDUFB7-G2A-FLAG, and NDUFB7-G2A-CtoS-FLAG**.

The intracellular localization of these four proteins was assessed by an immunofluorescence analysis of COS-1 cells transfected with cDNA coding these four proteins using an anti-FLAG antibody. Hoechst, MitoTracker Red, and TGN46-EGFP were used as organelle markers for the nucleus, mitochondria, and Golgi apparatus, respectively. Experiments were repeated 3 times and similar results were obtained. Representative data are shown. Abbreviation used: PC, phase contrast image.

**C: Line profile analysis of the intracellular localization of NDUFB7-G2A-FLAG and NDUFB7-G2A-CtoS-FLAG.**

The results of a line profile analysis are shown. In the line profile analysis, corresponding line-scan graphs of the relative fluorescence intensities of green anti-FLAG or TGN46-EGFP fluorescence (green line) and red MitoTracker red or anti-FLAG fluorescence (red line) along the white line indicated in the merged images are shown.

**Supplementary Figure S9**

A. Analysis of protein N-myristoylation of MIC19-FLAG, MIC19-CtoS-FLAG, MIC19-G2A-FLAG, MIC19-G2A-CtoS-FLAG, NDUFB7-FLAG, NDUFB7-CtoS-FLAG, NDUFB7-G2A-FLAG, and NDUFB7-G2A-CtoS-FLAG expressed in transfected COS-1 cells by metabolic labeling.

cDNAs encoding C-terminally FLAG-tagged proteins were transfected into COS-1 cells and cells were labeled with a myristic acid analog. The expression of proteins was evaluated by Western blotting using an anti-FLAG antibody (left panel). Protein N-myristoylation was evaluated by metabolic labeling followed by click chemistry, as described in the Methods (right panel).

B. Raw images for Supplementary Fig. S9A.

**Supplementary Table S1.**

**
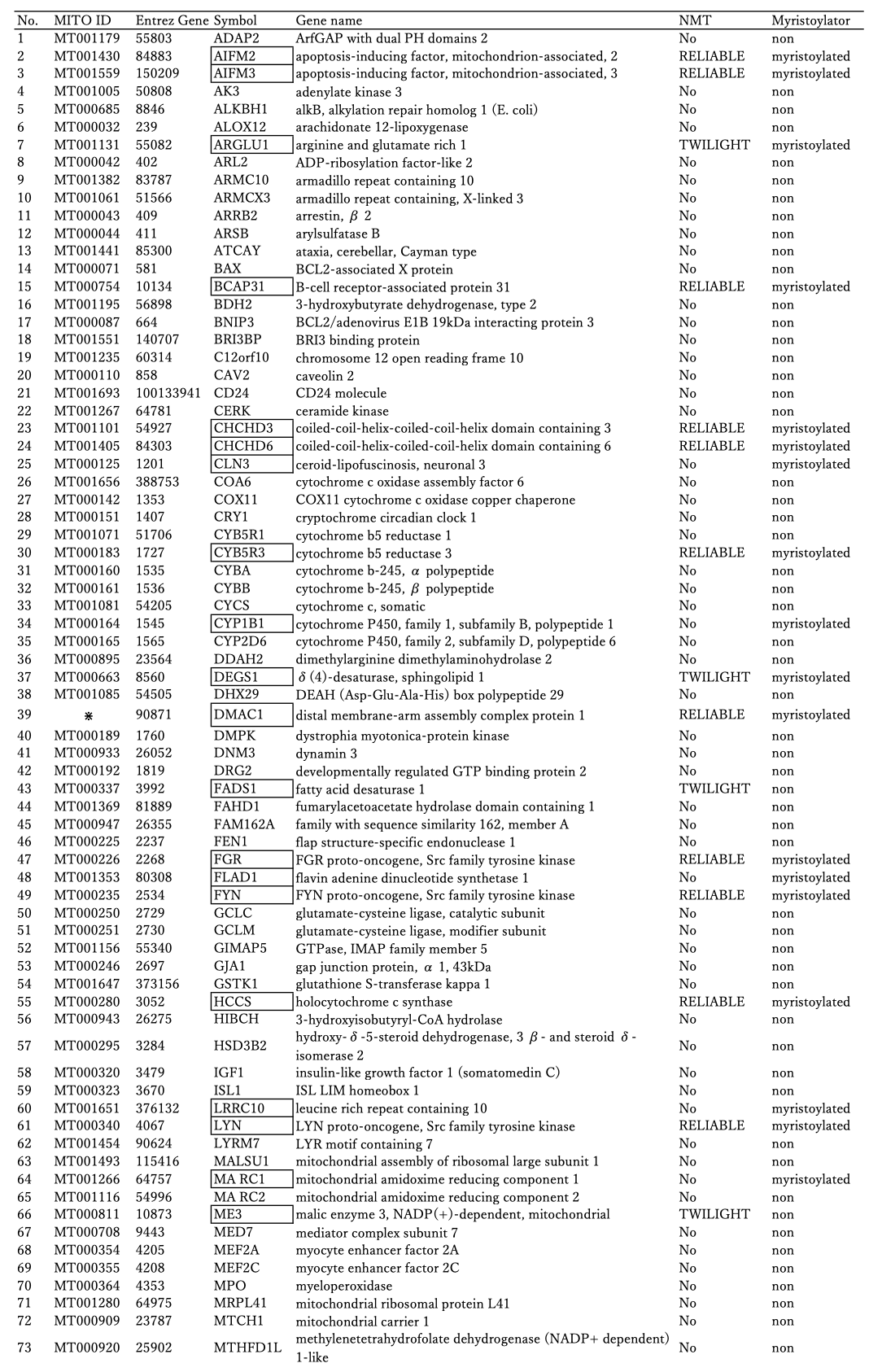
**

**
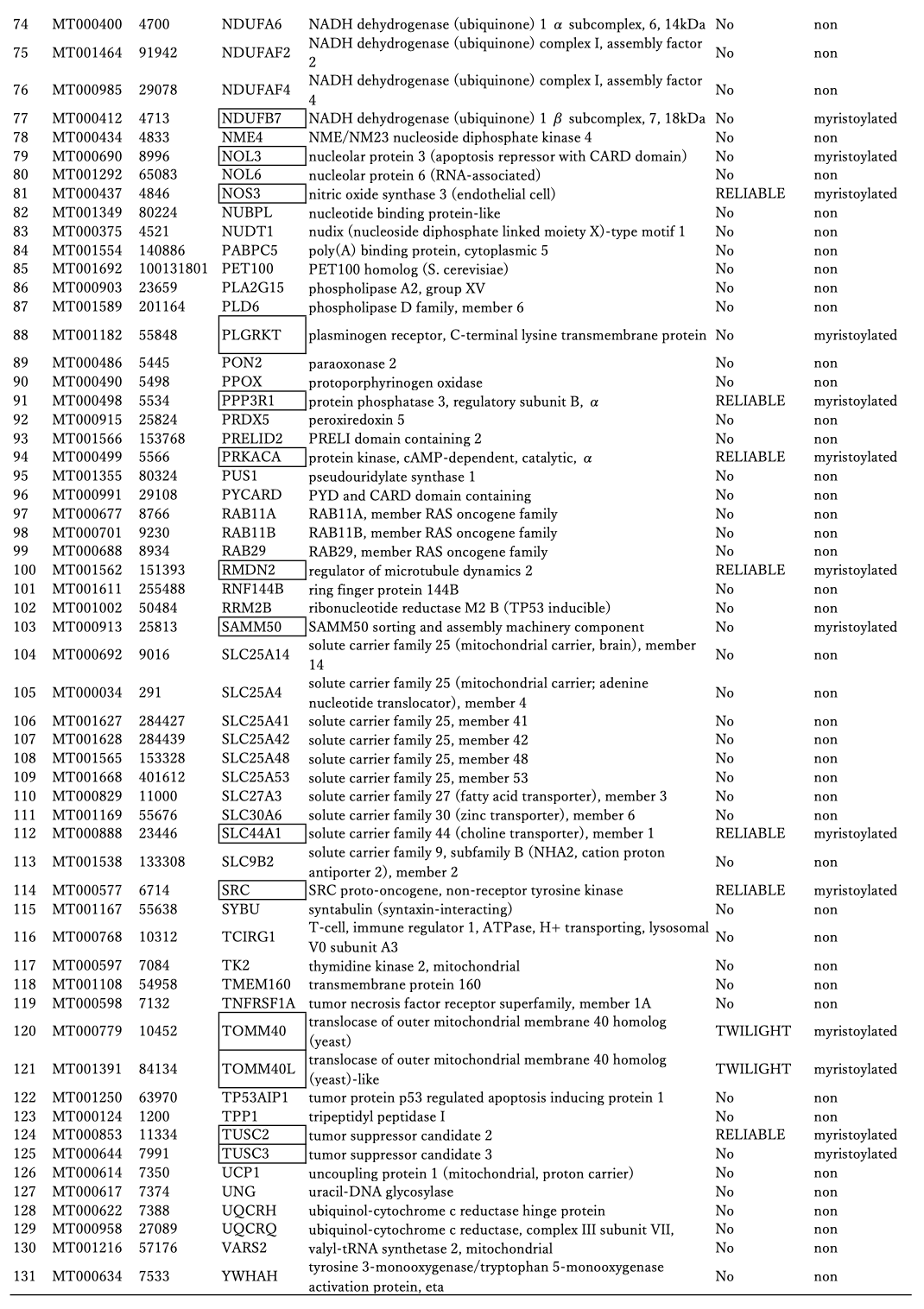
**

The results of the prediction for protein N-myristoylation of 131 gene products with N-terminal Met-Gly motifs listed in the MitoProteome protein database using two prediction programs, The MYR Predictor (NMT) and Myristoylator. The positively predicted genes were boxed. ※: The gene *DMAC1/TMEM261* is not listed in MitoProteome protein database. However, it was reported to be a mitochondrial protein in the report published in 2016 (Ref.23). Therefore, we added this gene in the list.

**Supplementary Table S2.**

The information about the 13 genes analyzed in this study was summarized.

**Supplementary Table S3.**

The nucleotide sequences of oligonucleotides used to construct pcDNA3-X(N10)-tGelsolin-FLAG

**Supplementary Table S4.**

The nucleotide sequences of oligonucleotide primers used for PCR

**Supplementary Table S5.**

The nucleotide sequences of oligonucleotides used for mutagenesis

**Supplementary Table S6.**

Strategies for the construction of pcDNA3 plasmids including various cDNAs by PCR

**Supplementary Table S7.**

Strategies for the construction of pcDNA3 plasmids including cDNA coding MIC19 and NDUFB7 mutants by site-directed mutagenesis
